# Probiotics and Postbiotics as Substitutes of Antibiotics in Farm Animals: A Review

**DOI:** 10.3390/ani11123431

**Published:** 2021-12-01

**Authors:** Daria Zamojska, Adriana Nowak, Ireneusz Nowak, Ewa Macierzyńska-Piotrowska

**Affiliations:** 1 Polwet-Centrowet Sp. z o.o., M. Konopnickiej 21, 98-100 Lask, Poland; emacierzynska@gmail.com; 2 Department of Environmental Biotechnology, Lodz University of Technology, Wolczanska 171/173, 90-530 Lodz, Poland; 3 Faculty of Law and Administration, University of Lodz, Kopcinskiego 8/12, 90-232 Lodz, Poland; inowak@wpia.uni.lodz.pl

**Keywords:** broiler, piglet, pig, bovine, lactic acid bacteria, probiotic, postbiotic, antibiotic, farm animal

## Abstract

**Simple Summary:**

Breeders are searching for methods to protect farming animals against diseases caused by pathogenic bacteria. The easiest way to fight bacteria is to use antibiotics. Unfortunately, their abuse results in the presence of bacteria resistant to the most commonly used antibiotics in the environment. The restrictions on the use of antibiotics have forced the search for natural and safe ways to protect animals. It has been shown that the use of probiotics based on lactic acid bacteria may have a positive effect on the growth and use of feed by broilers, on the stabilization of the intestinal microbiota of chickens and pigs, and in the prevention of mastitis in dairy cows. The use of probiotics (live, nonpathogenic microorganisms) and postbiotics (inanimate bacteria, cell components or post-fermentation by-products) reduces the occurrence of pathogens in large-scale farms.

**Abstract:**

Since 2006, the use of growth-promoting antibiotics has been banned throughout the European Union. To meet the expectations of livestock farmers, various studies have been carried out with the use of lactic acid bacteria. Scientists are trying to obtain the antimicrobial effect against the most common pathogens in large-scale farms. Supplementing the diet of broilers with probiotics (live, nonpathogenic microorganisms) stabilized the intestinal microbiota, which improved the results of body weight gain (BWG) and feed intake (FI). The positive effect of probiotics based on lactic acid bacteria has been shown to prevent the occurrence of diarrhea during piglet weaning. The antagonistic activity of postbiotics (inanimate bacteria, cell components, or post-fermentation by-products) from post-culture media after lactobacilli cultures has been proven on *Staphylococcus aureus*—the pathogen most often responsible for causing mastitis among dairy cows. The article aims to present the latest research examining the antagonistic effect of lactic acid bacteria on the most common pathogens in broilers, piglets, pigs, and cow farms.

## 1. Introduction

The invention of antibiotics is considered to be one of the greatest discoveries of the twentieth century and made it possible to control many diseases, but with their “overnormative” use, new threats appeared, including antibiotic resistanceto both human and veterinary medicinal products for animals [1]. It is obvious that the consumption of meat and meat products from animals fed with antibiotics (including their residues) is undoubtedly harmful to the health of the latter, and even more so to people, because even in a low concentration and regardless of the period time, it is not physiologically indifferent [2,3,4,5]. In other words, in animal husbandry, antibiotics should only be used for therapeutic purposes and not, *inter alia*, for reducing falls due to the crowding of animals in a small area, improving their condition, or stimulating growth and development. Thus, it is unacceptable to legally legalize the maximization of the fattening of farm animals through the use of antibiotics solely for strictly economic reasons [5]. The development of agriculture has led to the introduction of intensive livestock to satisfy parts of society around the world. Unfortunately, intensive industrial breeding increases density on farms and promotes the development of various diseases [6,7]. Crowded livestock production conditions facilitate the transmission of zoonotic pathogens, such as parasites, fungi, viruses, and bacteria among individuals [8,9].

In 2019, the EU produced 43.5 million tons of meat, which was around 10% less than in 2018. From the number, approximately 52% came from pigs (22.8 million tons), 6.9 million tons were bovine meat, and 13.3 million tons were poultry. In the same year, the EU produced 158.2 million tons of raw milk. The European Union bred 143 million pigs, and 77 million cattle were also bred. The main producer of poultry meat in the EU was Poland (2.6 million tons) [10].

The sales of pharmaceutical forms of drugs in 31 countries accounted for 87.7% of the total sales of veterinary products: premixes accounted for 26.9%, oral powder for 9%, and solutions for 51.8%. The sales of intramammary preparations amounted to 0.6%. In the years 2011–2018, a decrease in sales by 34.6% was observed in 25 countries. Sales of tetracyclines (30.7%), penicillins (28.8%), and sulfonamides (8.4%) in mg/PCU accounted for 67.9% of total sales in 2018 [11]. One experiment showed an increased productivity of chickens after the administration of subtle doses of antibiotics, such as chlorotetracycline, virginiamycin, and amoxicillin. In animals fed with antibiotics, the feed conversion ratio (FCR) decreased, but in the feces samples, heterotrophic bacteria with antibiotic resistance were detected [12]. The overuse of antimicrobial agents in animal husbandry causes more and more frequent occurrences of pathogenic bacteria resistant to popular antibiotics [13].

In the review, the positive effect of the use of probiotics on achieving the balance of intestinal microorganisms and the antibiotic-like effect in inhibiting the growth of pathogenic microorganisms found in slaughter farms in chickens, pigs, piglets, and cattle is described. According to the definition used by The International Scientific Association for Probiotics and Prebiotics (ISAPP), a probiotic is a live, non-pathogenic microorganism with a positive effect on the host [14]. According to Williams (2010), microorganisms classified as probiotics are most often lactic acid bacteria (LAB), but also *Bacillus cereus* and *Enterococcus faecalis* [15]. Probiotic bacteria, in particular LAB, are used to obtain fermented dairy products, e.g., Greek yogurt (*S. thermophilus* ACA-DC 26), feta cheese (*L. plantarum* ACA-DC 2640) [16], kefir [17,18], and fermented vegetables, e.g., sauerkraut [19,20]. Consuming probiotics shows a health-promoting effect for humans [21,22]. Likewise, probiotics protect food against the development of pathogens and spoilage [22,23]. The review also describes the positive effects of postbiotics on the animals’ gut microbiome.

**Figure 1:**
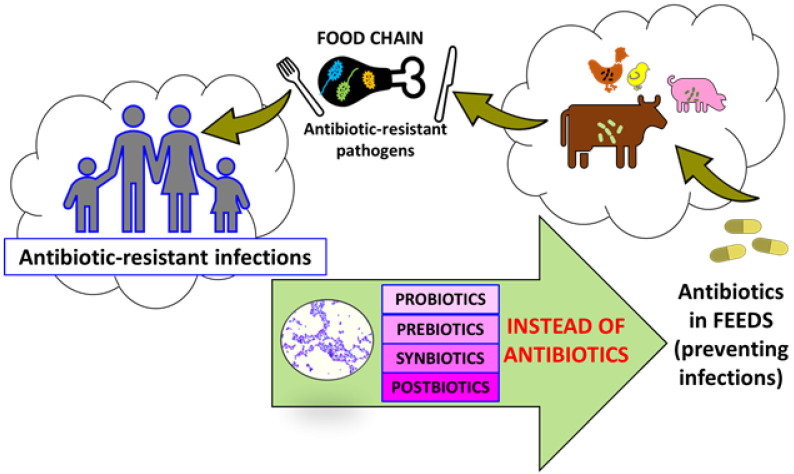
Effect of antibiotics application in animal husbandry.

The latest ISAPP definition says that postbiotics are inanimate microorganisms or bacterial cell components and that post-fermentation by-products such as lactic acid and short chain fatty acids (SCFA) positively affect the host [14]. According to Pandey et al. (2015), a synbiotic is a food component or dietary supplement consisting of an appropriate combination of a probiotic and a prebiotic [24]. The ISAPP gave a more precise definition of synbiotic. Synbiotic causes a positive effect on the health of the host, and it is a mixture of microorganisms and nutrients used by the host’s microorganisms [25]. A prebiotic is a substance used by microorganisms that has a positive effect on its growth [14]. In the cited example, the prebiotic was the polysaccharide inulin [26] added to the probiotic mix of LAB bacteria [27] (Figure 1).

This article discusses the latest research on the effects and benefits of LAB and their metabolites on the intestinal microbiota of the most common farm animals: poultry, bovines, and pigs. The review covers the newest literature on the application of probiotics and postbiotics in nutrition and in improving the welfare of farm animals. In addition, the future legal regulations regarding antibiotics in the European Union are discussed.

## 2. The Welfare Improving Poultry Farming

The most common pathogen in the poultry gastrointestinal tract in broiler breeding is the *Salmonella* species. Due to the limitation of the use of antibiotics, research is conducted with the use LAB bacteria in order to limit the colonization of the intestines by *Salmonella* sp. Wang et al. vaccinated hatched chicks with a strain of *Lactiplantibacillus plantarum* LTC-113. Studies have shown protection against *Salmonella* Typhimurium by reducing gut colonization and stabilizing tight junction gene expression in intestinal epithelial cells among treated chickens. In the control group, a *Salmonella* infection disrupted the intestinal epithelial barrier [28]. 

In contrast, another study reported that orally administered *Lactobacillus johnsonii* reduced intestinal colonization by *Salmonella* and *Clostridium perfringens* [29]. In another variant, the probiotic was given combined with two strains: *Ligilactobacillus salivarius* and *Enterococcus faecium*. The probiotic reduced intestinal colonization by *Salmonella* Enteritidis and did not cause weight loss or damage to the gastrointestinal mucosa [30]. In another experiment, the use of the *E. faecium* probiotic in feed increased egg weight, serum FSH (follicle-stimulating hormone) levels, and decreased *Bacteroidetes* (phylum) in low reproductive individuals [31]. *Salmonella* enters the bloodstream and then goes to the liver and spleen through damage to the intestinal barrier, which has been confirmed in many studies [32,33,34].

The administration of LAB bacterial cell membrane extracts to chickens reduced the *Salmonella* Enteritidis infection. Considerable protection of the intestinal epithelium against the effects of infection was evident [32]. The next experiment was very similar, however, a commercial probiotic based on LAB with proven efficacy against *S.* Enteritidis was used for treatment. The study aimed to understand the effect of a probiotic on intestinal colonization and intestinal permeability in infected chickens. The results were very promising. After infection of the control group with *Salmonella* pathogens, heterophilia and lymphopenia were observed as well as an increase in basophils and eosinophils compared to the chickens treated with a probiotic based on LAB bacteria. In the control group, increased intestinal permeability was also found [33]. In another experiment, the probiotic *Bacillus subtilis* C-3102 was used to control *S. enteric* serovar *enteritidis* LM-7. The specific pathogen-free chicks became infected by administering an appropriate dose of *Salmonella* in the food. Supplementation with *B. subtilis* feed reduced *Salmonella* infections and may accelerate the clearance of pathogens in the liver, cecum, and spleen of chicken farms [34].

Han et al. (2017) orally administered *Pediococcus acidilactici* mutants to chickens, modulating the microbiota and reducing the number of *Salmonella enterica* serovar Gallinarum, which is often found in the poultry industry. The study proved the antimicrobial activity of *P. acidilactici*. After treatment of the cultures with proteinase, the antimicrobial activity decreased, which may suggest the production of proteinaceous substances such as bacteriocin by *P. acidilactici* [35].

Another common pathogen on farms is *Campylobacter* sp. The pathogen is rapidly transmitted on poultry farms via the fecal–oral route. The study of Ščerbová et al. (2016) assessed the inhibition spectrum of various enterocins against *Campylobacter* sp. Enterocins are protein substances with antibacterial activity metabolized mainly by enterococci. The isolated strains from poultry farming were divided into two groups, *Campylobacter jejunum*, and *Campylobacter coli*. Interestingly, the strains showing resistance to antibiotics displayed sensitivity to at least one of the nine eneterocins tested [36]. On the other hand, Razmyar et al. (2017) showed that *C. perfringens* secretes bacteriocins, which may be responsible for facilitating intestinal colonization and causing intestinal inflammation by this pathogen [37]. Supernatants from *Lactobacillus acidophilus* NCFM, *Lactobacillus crispatus* JCM 5810, *Lactobacillus gallinarum* ATCC 33199, and *Lactobacillus helveticus* CNRZ32 cultures inhibited the growth of *C. jejuni in vitro*. After the analyses, the substance responsible for limiting the growth of *C. jejuni* was partly lactic acid. Subsequently, in *in vivo* studies, LAB was administered to broilers on the day of hatching, on the fourth day after hatching, and 14 days after hatching, challenged with *C. jejuni* F38011. Each of the four strains limited the colonization of the pathogen. It was most effective in limiting the colonization of *L. crispatus* JCM 5810 [38].

Another pathogenic bacterium on chicken farms is avian *E. coli* (APEC). Birds infected with APEC show macroscopic changes in air sacs and lungs [39]. In the experiment of Li et al. (2021), *Lactobacillus animalis* (ATCC 35046), *Lactobacillus reuteri* (ATCC 2837), and *Lactobacillus rhamnosus* (ATCC 23272) strains were injected into the eggs to inhibit APEC infection. However, no differences were noted in APEC-like strains infection in the probiotic-administered group and control group [40]. This result was completely different from that obtained in the experiment in which the probiotic mix with *B. subtilis*, *Clostridium butyricum*, and *L. plantarum* was used. The applied probiotic lowered the *E. coli* index in infected chickens. The positive effect of the probiotic on the modulation of the intestinal microbiota of broilers has also been proven [41].

The protection of poultry farms against microbes is one of the most important factors in good breeding. However, it is also an important factor to improve European indicators for weight gain and feed consumption. The study of Kierończyk et al. (2017) aimed to test the effect of nisin on growth efficiency, morphological parameters, the activity of digestive enzymes, the digestibility of nutrients, and the effect on intestinal morphology in chickens. It was noticed that supplementing the diet of chickens with nisin, which is a bacteriocin used in the preservation of food products [42], improved the body weight gain (BWG), feed conversion ratio (FCR), and feed intake (FI) indexes [43]. According to Hsu et al. (2004), nisin is a cyclic polypeptide that contains 34 amino acids. The lactic acid bacteria of the genus *Lactococcus lactis*, carries out the fermentation process and, in addition to lactic acid, produces the bacteriocin nisin. Nisin is a natural antibiotic against Gram-positive bacteria [44,45]. Research has indicated that nisin can be used as a growth simulator without adversely affecting the bird’s metabolism or immunity levels [28]. According to Kierończyk et al. (2020), nisin can be considered as a new and natural growth promoter. It improves digestibility and feeds conversion. By limiting the multiplication of pathogenic bacteria, nisin has a positive influence on the modulation of the intestinal microbiota. In terms of its antibacterial properties, it is similar to monensin, an antibiotic of the coccidiostatic type [46].

The intestinal microbiota was modulated not only with probiotics but also with plant feed additives. Wang et al. (2021) showed a positive effect of dietary purslane in the experiment. Purslane (*Portulaca oleracea* L.) is an edible wild vegetable with medicinal properties. The use of purslane in the feed increased the level of *Lactobacillus* and lowered *Escherichia/Shigella* in the digestive tract of broilers. The growth of beneficial bacteria in chicken intestines may promote high body weight gain [47]. Another study by Liang et al. (2021) applied traditional Chinese medicine based on medicinal plants combined with probiotics—a mix of *B. subtilis* and *L. acidophilus*. In the treatment of *E. coli*-infected chickens, the mix inhibited the survival level of *E. coli*, reduced the rates of diarrhea and mortality, improved body weight gain, and relieving pathological changes in the intestines and liver were observed [48].

In another study confirming the beneficial effect of LAB on the intestinal microbiota of fattening chickens conducted by Śliżewska et al. (2020), the effects of three variants of synbiotics were compared with two variants of a commercial probiotic on the chicken’s performance. The synbiotics were three combinations containing the following strains: *L. plantarum* ŁOCK 0860, *L. reuteri* ŁOCK 1092, *L. pentosus* ŁOCK 1094, *Saccharomyces cerevisiae* ŁOCK 0119, *L. rhamnosus* ŁOCK 1087, *L. paracasei* ŁOCK 1091, and 2% inulin (prebiotic). Commercial probiotics included BioPlus YC (*Bacillus licheniformis* DSM 5749, *B. subtilis* DSM 5750) and Cylactin (*Enterococcus faecium* NCIMB 10415). The positive effect of synbiotics on the performance of fattening chickens and the balance of the intestinal microbiota was demonstrated. The number of beneficial microorganisms such as *Bifidobacterium* sp. and *Lactobacillus* sp. increased in the intestines, and the number of pathogenic bacteria such as *Clostridium* sp. and *E. coli* in the intestines and animal excretions decreased. The change in the gut microbiome increased the levels of lactic acid and SCFA (short chain fatty acid). This is another study confirming the beneficial effect of LAB on the intestinal microbiota of fattening chickens [27].

The Gram-negative bacteria *Gallibacterium anatis* is responsible for the decrease in the number of eggs laid by causing infections of the genital tract of hens and contributes to increased mortality [49,50]. A study conducted by Zhang et al. (2021) showed the antagonistic activity of the supernatant after the culture of *Leuconostoc mesenteroides* QZ1178 (a species of lactic acid bacteria) was used against *G. anatis* strains *in vitro*. The antagonistic effect was decreased upon increasing the pH. After analysis of the supernatant, *L. mesenteroides* QZ1178 mainly produced lactic acid (29 mg/mL) and acetic acid (7 mg/mL), which are probably responsible for its antibacterial properties [51]. The above information is summarized in Table 1.

## 3. Prevention of the Effects of Piglet Weaning Based on the Use of LAB

The critical moment in breeding is weaning the piglets from sow on day 28. Pigs are very sensitive to changes in their living environment. This is a very stressful time for piglets, causing destabilization of the intestinal microbiota. During this time, digestive disorders, diarrhea, growth retardation, and increased mortality occur [61,62,63,64]. The most common pathogens affecting intestinal disorders and damage to intestinal villi are *E. coli*, *C. perfringens*, *Salmonella* Choleraesuis, and *Salmonella* Typhimurium. In the case of infection, the permeability of fluids to the intestinal lumen increases and diarrhea develops. There is also an increase in pH, which prevents the multiplication of LAB [65].

Antibiotics are used to improve the intestinal microbiota and reduce the occurrence of diarrhea caused by weaning. The overuse of antibiotics has resulted in the emergence of pathogenic bacteria resistant to the basic antibiotics used in farming. Moreover, in the case of pig farms, research on the use of LAB to improve the intestinal microbiota was started [66].

Verso et al. (2018) isolated 595 pure cultures of bacteria from the small and large intestines from pre and post-weaned piglets. The selected bacteria were antagonistics to pathogens and were capable of producing antimicrobial compounds. First, the activity against pathogens *E. coli* MC4100, *S.* Choleraesuis ATCC 29628, and *Listeria innocua* HPB13 was tested using the Double-Agar-Layer Technique Method. At total of 51.1% of all strains showed antimicrobial activity and passed the next test to investigate the production of antimicrobial compounds. For this, the pH of the supernatant was neutralized with 1M NaOH. Activity against previous pathogens and *Staphylococcus aureus* ATCC 6538, *Enterococcus faecalis* ATCC 27275, *Listeria monocytogenes* LSD530, and *S.* Enterica ATCC 8387 was tested by the Agar-Well Diffusion Method. Loss of activity of some supernatants after protease treatment may indicate the presence of bacteriocin-like substances. These studies confirmed the ability of some LAB strains of the species *L. salivarius* and *Lactobacillus delbrueckii* subsp. *lactis* isolated from the digestive tract of pigs to inhibit the growth of potential pathogens by the production of organic acids in combination with bacteriocin-like proteins [67]. In another experiment, piglets were fed with an immunobiotic feed based on okara fermented soy milk with *L. delbrueckii* subsp. *delbrueckii* TUA4408L. The beneficial intestinal microbiota improved, the amount of *Lactobacillus* and *Lactococcus* increased, and the immunity also increased. The piglets showed better meat quality and growth performance [68]. In addition, the use of *L. delebureckii* CCTCC M 207,040 as a diet supplement by Chen et al. (2021) improved the gut structure resulting in increased gut integrity in lipolisaccharides (LPS)-challenged piglets. LPS stress induced an increase in the depth of the crypts in the jejunum and ileum. However, the use of *L. delebureckii* dietary supplementation reduced crypt depth compared to the non-challenged controls and LPS-challenged. Moreover, the TLRs-Btk-Nrf2 signaling pathway, which mediates oxidative stress, was mitigated [69]. Similar results were obtained in weaned piglets with *C. butyricum* ZJU-F1 and *B. licheniformis*. The intestinal permeability was reduced, and the digestibility of nutrients and the expression of antimicrobial peptides in the ileum improved [70]. In another experiment, Sobrino et al. (2021) isolated *L. salivarius* MP100 from sow’s milk and gave inoculated feed to pregnant sows and piglets. MP100 showed antagonistic activity against the indicator bacteria: *C. perfringens* MP34, *E. faecalis* MP42, *S. aureus* MP83, *Streptococcus suis* MP205, *Trueperella pyogenes* MP214, *E. coli* MP73 (F4) and MP77 (F18), *S.* Typhimurium MP55, and *Klebsiella pneumoniae* MP87. The use of a potential probiotic resulted in a microbiological and biochemical improvement in the gut environment [71].

The *Lactobacillus gasseri* LA39 and *Limosilactobacillus frumenti* strains produce the substance gassericin A, which is a bacteriocin. A characteristic feature of this protein is that it binds to the intestinal epithelium of the host and makes it resistant to diarrhea in weaned piglets. Gassericin A bound to Keratin 19 in the plasma membrane of the intestinal epithelium increased the absorption of fluid from the intestine and reduced its secretion. The early weaning of piglets aims to shorten the slaughter cycle and improve the reproduction of the sows. In the experiment, fecal microbiota were taken from healthy Congjiang miniature pigs (a Chinese native pig breed) and administered orally to a commercial Landrace × Yorkshire (LY) pig, which shows frequent diarrhea after weaning. This treatment made the LY immune to stress-related diarrhea at weaning. The *L. gasseri* LA39 and *L. frumenti* strains can be an alternative to antibiotics in the prevention of diarrhea during increased stress in piglets [72]. In one study, the administration of *L. salivarius* (strains 144 and 160) to suckling piglets early in life resulted in an increase of the amount of *Lactobacillus* in the gastrointestinal tract and a reduction in the number of *Bacteroides* and *Fibrobacter*. The incidence of diarrhea during the most stressful time of weaning also decreased. The conclusions from the conducted research indicated the use of supplementation with *L. salivarius* 144 isolated from healthy pigs with a high BMI (body mass index), as it had a beneficial effect on increasing the height of intestinal villi, which influenced the pigs’ growth efficiency. The same strain showed a reduction in the amount of *Clostridium* sp. in the feces [73].

Another way of administering LAB strains to pigs was to use of microcapsulation, i.e., administering LAB strains in sealed gelatin–alginate capsules. This method protects microorganisms against unfavorable conditions in the digestive tract. In the study of Le et al. (2019), the strains were isolated from traditional Vietnamese fermented yogurt. After the bacteria had multiplied, they were centrifuged, the supernatant was removed, and the remaining biomass was encapsulated. The *L. plantarum* SC01 strain showed antagonistic activity against *E. coli*, *S. aureus*, *B. subtilis*, *Salmonella* sp., and *L. monocytogenes*. It has been shown that the concentration of 2.5% (*w*/*v*) of alginate and 6% (*w*/*v*) of gelatin increases the production of a highly active compound that inhibits pathogens by LAB [74]. On the other hand, Pupa et al. (2021) used spray drying microencapsulation of *L. plantarum* 22F. The application of this method as well as alginate and chitosan usage for the production of capsules extended the viability of probiotic bacteria. The increased performance of pigs after microencapsulated probiotic supplementation was comparable to the use of live bacterial cultures. The alive bacteria: *L. plantarum* (strains 22F and 25F) and *P. acidilactici* (strain 72N) were administered as potential probiotic supplements. The best effect in reducing pathogenic intestinal strains (*Enterobacteriaceae*) and modulating lactobacilli in the intestinal tract was obtained with the use of *P. acidilactici* 72N. The administration of the probiotics to young animals resulted in the improvement of intestinal integrity, elongation of intestinal villi in the jejunum, the appearance of microorganisms positively influencing the intestinal microbiome, and improved growth of individuals in the rearing cycle [75].

The weakening of the animals also occurs during pregnancy and the lactation of sows. Wang et al. (2014) isolated *L. johnsonii* XS4 from the gastrointestinal mucosa of healthy laboratory pigs. In previous studies, they demonstrated a high resistance of *L. johnsonii* XS4 to hydrochloric acid and bile salts and an antagonistic effect against the most common pathogens in culture (*S. aureus*, *E. coli*, *S.* Enterica). The sows were supplemented with freeze-dried *L. johnsonii* XS4 from day 90 of gestation to day 25 of lactation. It was noted that the supplementation resulted in an increase in the number of piglets weaned from the sow and an increase in litter weight by 14.45% compared to the control. In the supplemented group, there was a lower loss of backfat during lactation than in the control group, but it was not statistically significant. The obtained results indicate a positive effect on the production efficiency of the sows and the obtained litter. *L. johnsonii* XS4 has been presented as a promising alternative to the use of antibiotics in feed [76]. In another study, 295 LAB strains were tested, and three strains (*Limosilactobacillus reuteri* P7, *Lactobacillus amylovorus* P8, and *L. johnsonii* P15) with high growth-inhibitory activity for enterotoxigenic *E. coli* K88 were selected. The strains had a positive effect on the reproductive performance of sows and the growth of weaned piglets and reduced the occurrence of diarrhea [77].

In contrast, supplementation with *E. faecium* DSM 7134 increased food digestibility, gross weight, and gross energy. Moreover, in this case, the supplementation affected the sow’s litter. The mortality of weaned pigs decreased, and weight gain of the piglets was noticeable. In this experiment, a reduced number of *E. coli* in feces was demonstrated after the piglets were weaned [78].

The administration of probiotic mixes to the sow, in this case, composed of *L. delbrueckii* subsp. *bulgaricus*, *L. rhamnosus*, *L. acidophilus*, *L. plantarum*, *Streptococcus salivarius* subsp. *thermophilus*, *Bifidobacterium bifidum*, *E. faecium*, *Candida pintolopesii*, and *Aspergillus oryzae* also gave satisfactory results. In this study, no diarrhea occurred in either the sows or the piglets. An increased concentration of acetic, propionic, and butyric acids in the feces was shown in piglets supplemented with the mix. However, in this case, the supplementation of the sows did not affect the litter weight, but there was a visible change in the gut environment of the piglets [79]. In the case of using only the dietary supplementation *L. plantarum* CAM6 in sows, it had a positive effect on body weight and reduced the occurrence of diarrhea in the offspring. The nutritional value of milk in sows improved [80]. The application of a diet with *L. plantarum* JL01 for weaned piglets resulted in better digestion and absorption of fats in the cecum and mediated the metabolism of tryptophan [81,82].

The interest of scientists was also aroused by the influence of a probiotic diet on bacteria present in the air. The effect of administering feed supplemented with *E. faecalis* CICC 23,215 in a piglet house over 60 days of airborne bacterial communities in the house was investigated. Air and feces samples were tested. The enrichment of air and feces with *Lactobacillus* species was shown. *E. faecalis* reduced the abundance of Proteobacteria, *Acinetobacter* sp., *Escherichia* sp., and *Shigella* sp. [83].

In another study by Wang et al. (2021), the effect of feeding with feed co-fermented by *B. subtilis* CW4 and *E. faecium* CWEF on lactating sows and newborn piglets was investigated. It was shown that the quality of the sows’ milk was improved, which resulted in an increased weight gain in the piglets. There was a reduction in the incidence of constipation in sows and diarrhea in piglets, which was related to the modulation of the intestinal microflora. The sows showed better immunity and performance compared to the control [84]. Another experiment used the positive effects of *Bacillus coagulans*, oregano oil, and benzoic acid on the health, physiological, and physical condition of the piglets. An increased weight gain with a reduced feed ration was obtained in the diet supplemented with benzoic acid and *B. coagulans*. The addition of a third component to the diet—oregano oil—resulted in an increased number of bifidobacteria in the caecum and a decreased *E. coli* population in the cecum. Both the supplementation of the two-component and three-component diets positively influenced intestinal integrity, immunity, and physical condition of piglets in non-antibiotic breeding [85]. Similarly, in the study by Fu (2021), the use of *B. coagulans* and yeast hydrolysate in weaned piglets improved the intestinal barrier function, which resulted in better weight gain of the piglets. These two options can be used as alternatives to antibiotic growth promoters [86].

Not only LABs can be used as potential probiotics to prevent diarrhea. Swine commensal *E. coli* strains are bactericidal and compete in the environment with pathogenic porcine strains of *E. coli*. A reduced susceptibility of commensal *E. coli* to the 34 bacteriocin monoproducers was demonstrated compared to pathogenic *E. coli*. Finally, *in vitro* and *in vivo*, three potential probiotic *E. coli* were selected that could be candidates for the prophylaxis of post-weaning diarrhea [87]. The above information is briefly summarized in Table 2.

## 4. Prevention of Udder Infections in Cattle with Particular Emphasis on Bacterioci Like-Substance

Inflammation of a cow’s udder, another name mastitis, is caused by physical trauma, chemical irritation, or bacterial infection. Mastitis can be identified in two types: clinical and subclinical. Clinical mastitis manifests itself with local and systemic symptoms. Redness, swelling, pain in the udder area, decreased appetite, increased temperature, reduced milk production, and a change in milk compounds are visible [90]. Subclinical mastitis does not alter the udder. Abnormal changes in milk are visible, such as an increased population of bacteria, a change in quality and compounds, reduced milk production, and an increased number of somatic cells [91]. Inflammation of the udder tissue has a negative impact on economical milk production and the animal’s welfare due to pathologies causing edema, swelling, pain, inflammation, or udder fibrosis [92] and to a reduced reproductive efficiency [93]. Disease control is hindered by the causes of multifactorial occurrence and involving a large number of pathogens [94]. The main pathogens inducing mastitis are *S. aureus*, *Streptococcus uberis*, and *Streptococcus dysgalactiae* [95,96,97] and more often isolated strains of *E. coli* [98]. The treatment consists of identifying the pathogen and then administering an appropriate intramammary antibiotic [99]. Due to the excessive use of antibiotics, bacterial resistance increases and treatment efficacy decreases, so there is a growing interest in replacement therapies without antibiotics [100].

One experiment reviewed a large bank of engineered nisin modifications and discovered three new variants of nisin A M17Q, nisin A HTK, and nisin A T2L. These variants showed antibacterial activity against *S. aureus* strains associated with bovine mastitis. It has been shown to reduce the growth inhibition of commensal bacteria naturally occurring in milk, such as lactobacilli and lactococci. This is the next step in the development of substitutes for antibiotic therapies on farms [101].

Godoy-Santos et al. (2019) isolated the bovicin HC5 bacteriocin from the rumen bacteria *Streptococcus equinus* HC5. Bovicin HC5 is a lantibiotic with the ability to bind to lipid II in the cytoplasm such as nisin [102]. Other studies showed a bovicin HC5 bactericidal effect against *L. monocytogenes*, *Salmonella* Typhimurium, and some species of *Clostridium* and *Bacillus* [103,104]. The antibacterial effect of bovicin HC5 was tested in isolated strains from animals with mastitis. There were: *S. aureus* (99 strains), coagulated negative *Staphylococcus* sp. (CNS) (44 strains), *Streptococcus agalactiae* (71 strains), *Streptococcus bovis* (22 strains), *S. uberis* (20 strains), and *E. coli* (20 strains). Bovicin HC5 has inhibited the growth of most *Streptococcus* and *Staphylococcus* species. A total of 276 pathogenic isolates were tested. Some 18% of the isolates were not susceptible to the bacteriocin. None of the *E. coli* strains showed sensitivity to bovicin HC5. In the case of *S. aureus*, the pathogen most often responsible for the occurrence of mastitis in cows, as many as 95% of isolates showed the highest sensitivity to bovicin HC5 [102].

Due to the high costs of producing pure bacteriocins, other scientists decided to test the effect of a preparation containing live *Lactococcus lactis* DPC3147 cultures [105]. The effectiveness of the product was compared with the commercial antibiotic, Terrexine TM, which is used to treat cows with clinical and subclinical signs of mastitis. In cows with clinical symptoms, inflammation and/or malaise, clots, pathogens in milk, and poor milk production occurred. Cows infected with *S. aureus* and treated with biopreparation showed a cure rate of 45%, and cows treated with a commercial antibiotic showed a cure rate of 50%. The above data show that the biopreparation containing the bacteriocin lacticin 3147 produced by *L. lactis* DPC3147 shows comparable efficacy to the commercial antibiotic. Cows treated with the biopreparation showed an increased response of the immune system and a decrease in the number of somatic cells in milk. Five days after the injection into the replacement biopreparation, no DPC3147 cells were detected, which indicates that the animals quickly excrete the “live biopreparation”. The above experience allows us to limit the administration of antibiotics in the treatment of mastitis in the future and shorten the withdrawal time of treated animals [105].

On the other hand, one of the *in vitro* studies demonstrated the bactericidal effect of the supernatant after the *L. lactis* ssp. *lactis* bacterial culture was used against the most common occurring mastitis pathogen. There were obtained uncleaned bacteriocins from supernatant after bacterial culture and used on pathogens. In this experiment, Malvisi et al. (2016) confirmed the presence of the class I bacteriocin, nisin A, in the supernatant by liquid chromatography. The highest antagonistic activity had L. lactis LL11 and SL153 supernatants after bacterial culture. Malvisi et al. (2016) applied the supernatant containing nisin A to the bovine mammary epithelial cell line BME-UV1. The stimulation of the secretion of NAGase and the LZ antibacterial enzymes by the cells was visible without causing damage or adverse inflammatory reactions, and there was no damage to cell integrity [106].

One study investigated the antimicrobial activity of 13 specific bacteria isolated *in vitro* from a honey bee cultivation against mastitis pathogens. Nine types of Firmicutes were isolated: *Apilactobacillus kunkeei* Fhon2, *Apilactobacillus apinorum* Fhon13, *Bombilactobacillus mellis* Hon2, *Bombilactobacillus mellifer* Bin4, *Lactobacillus kullabergensis* Biut2, *Lactobacillus kimbladii* Hma2, *Lactobacillus helsingborgensis* Bma5, *Lactobacillus melliventris* Hma8, and *Lactobacillus apis* Hma11 and 4 types of Actinobacteria: *Bifidobacterium coryneforme* Bma6, *Bifidobacterium asteroides* Bin2, *Bifidobacterium* sp. Bin7, and *Bifidobacterium* sp. Hma3. Three of the tested oxalicin-resistant *S. uberis* were inhibited by the use of a combination of a honey-based medium and 13 specific bacteria. The same was observed in ampicillin and trimethoprim-sulfamethoxazole-resistant *E. coli* isolates. The study demonstrated that 13 specific bacterial symbionts in combination with the heather honey matrix showed an inhibitory effect on the growth of mastitis pathogens [107].

In one of the experiments carried out by Seon-Gyu Kim et al. (2019), synergistic inhibition of the growth of *S. aureus* KCTC 3881 by bacteriocin and bacteriophage was used. The bacteriocin was isolated from *L. lactis* CJNU 3001. For comparison, bacteriocin and bacteriophage activity were also tested separately. Pure bacteriocins showed antagonistic activity against *S. aureus* depending on the dose. Visible effects were obtained at concentrations of 50 and 100 AU/mL. The treatment of *S. aureus* with a dose of 1 MOI (multiplicity of infection) of the SAP84 bacteriophage showed a viable cell count of 5.7 Log CFU/mL. The combined action of phage (0.1 MOI) and bacteriocin (100 AU/mL) showed a reduction in the number of viable *S. aureus* cells to 3.3 Log CFU/mL. The combination of bacteriocin and bacteriophages may be a promising strategy to combat pathogens not only associated with mastitis in cattle [108].

One of the metabolites produced by LAB is lactic acid [38,109]. Chotigarpa et al. (2018, 2019) performed the time-killing analysis of rice gel with 5% (*v*/*v*) lactic acid on *E. coli* ATCC 25,922 and *E. coli* field strains (teat skin samples from healthy dairy cows after washing udder) in the time interval of 0 to 60 min. The study showed an inhibitory effect on the growth of *E. coli*, which means that created gel can reduce the number of pathogenic bacteria on the cows’ teats. The gel can be an alternative to the antibiotics used and in the prevention of mastitis. Additionally, the minimum inhibitory concentration (MIC) and minimum bactericidal concentration (MBC) of pure lactic acid were checked for *E. coli* strains. The MIC and MBC were 0.5% lactic acid [110,111].

The analysis of feces in four dairy cows showed the presence of the following *Lactobacillus* strains: *Lactobacillus gasseri*, *Limosilactobacillus reuteri*, and *Ligilactobacillus salivarius*. *In vitro*, the supernatants of these strain cultures showed bactericidal activity against *Escherichia coli* O157:H7, *Mycobacterium avium* ssp. *paratuberculosis*, and the *Salmonella* species (*Salmonella enteritidis*, *Salmonella typhimurium*, and *Salmonella* Dublin). They also showed a low risk of lateral transfer of antibiotic-resistant genes despite showing resistance to streptomycin (*L. gasseri*) and kanamycin (*L. salivarius*). In an *ex vivo* study, they showed adherence to bovine intestinal epithelium cells. The use of such fecal isolates may be a species-specific probiotic for cattle [112]. The above information is briefly summarized in Table 3.

## 5. Antibiotics—Future Legal Regulations

In order to counteract the phenomenon of antibiotic resistance and protect broadly understood public health, comprehensive actions, including legal ones, are necessary. In reference to the data provided by OECD (Organisation for Economic Cooperation and Development), it is estimated that about 700,000 deaths may be caused globally each year by AMR (antimicrobial resistance). Compared to a world with no AMR, the economic impact associated with current rates of AMR may reach about 0.03% of GDP in 2020 in OECD countries, 0.07% in 2030, and 0.16% in 2050. This would result in a cumulative loss of about USD 2.9 trillion (NB: in the quoted report, the amount ‘trillion’ means 1012—‘Council conclusions on the next steps under a One Health approach to combat antimicrobial resistance’ (2016/C 269/05), *Official Journal of the European Union* 23.7.2016) by 2050 [114]. Moreover, as shown by data published by the European Medicines Agency, the use of antibiotics in Europe is more than twice as high in the treatment of animals as in humans. From 2011 to 2014, the use of antibiotics in agriculture increased by 23% [115]. The basic legal act in the European Union in the field of the use of antibiotics, which will come into force on 28 January 2022, is Regulation (EU) 2019/6 of The European Parliament and of the Council on 11 December 2018 on veterinary medicinal products, and repealing Directive 2001/82/EC [116]. The above normative act will be directly applicable in all Member States, without the obligation to implement it into the national legal order.

According to the above-mentioned legal act, in Article 4 point 12 of the Regulation (EU), ‘antibiotic’ means any substance with a direct action on bacteria that is used for treatment or prevention of infections or infectious diseases. The antibiotic also fits into the broader definition adopted by the EU legislator, the so-called ‘antimicrobial’, which means any substance with a direct action on micro-organisms used for the treatment or prevention of infections or infectious diseases, including antibiotics, antivirals, antifungals and antiprotozoals) [116]. It is also worth recalling the broadest definition in terms of the analyzed regulation (*Vide* Article 4 point 1) [116]: ‘veterinary medicinal product’ means any substance or combination of substances which fulfils at least one of the following conditions:

(a) it is presented as having properties for treating or preventing disease in animals;
(b) its purpose is to be used in or administered to animals with a view to restoring, correcting, or modifying physiological functions by exerting a pharmacological, immunological, or metabolic action;
(c) its purpose is to be used in animals with a view to making a medical diagnosis;
(d) its purpose is to be used for euthanasia of animals.

Of key importance for the issue discussed is Art. 107 of the Regulation, which *expressis verbis* regulates the issues of the use of antimicrobial medicinal products. It shows, *inter alia*, that antimicrobial medicinal products cannot be used:

(a) antimicrobial medicinal products shall not be applied routinely nor used to compensate for poor hygiene, inadequate animal husbandry, or lack of care or to compensate for poor farm management;
(b) in animals for the purpose of promoting growth nor to increase yield;
(c) prophylaxis other than in exceptional cases for the administration to an individual animal or a restricted number of animals when the risk of an infection or of an infectious disease is very high, and the consequences are likely to be severe. In such cases, the use of antibiotic medicinal products for prophylaxis shall be limited to the administration to an individual animal only, under the conditions laid down in the first subparagraph.

A Member State may further restrict or prohibit the use of certain antimicrobials in animals on its territory if the administration of such antimicrobials to animals is contrary to the implementation of a national policy on prudent use of antimicrobials.

Antimicrobial medicinal products shall be used for metaphylaxis only when the risk of spread of an infection or of an infectious disease in the group of animals is high and where no other appropriate alternatives are available. Member States may provide guidance regarding such other appropriate alternatives and shall actively support the development and application of guidelines which promote the understanding of risk factors associated with metaphylaxis and include criteria for its initiation (*Vide* Article 107 Section 4) [116]. Antimicrobial medicinal products should not be used for prophylaxis other than in well-defined cases for the administration to an individual animal or restricted number of animals when the risk for infection is very high or its consequences are likely to be severe (*Vide* Article 107 Section 3) [116]. Member States should be able to allow the exceptional use of veterinary medicinal products without a marketing authorization where it is necessary to respond to Union-listed diseases or emerging diseases and where the health situation in a Member State so requires (*Vide* Section 26) [116]. It is worth emphasizing that the consequences of animal diseases, and at the same time the measures needed to combat them, can cause enormous damage to the entire population of animals, their owners, and thus the economy, and ultimately public health.

In order to fulfil the above-mentioned obligations as well as a whole range of other legal regulations regulated in the analyzed regulation Art. 107 Section 3, said Member States shall ensure that adequate financial resources are available to provide the staff and other resources necessary for the competent authorities to carry out the activities required by this Regulation [116].

The considerations carried out are only indicative, they undoubtedly prove that the widely understood processes of enactment, but above all the application of law, have a huge impact on the protection and guarantee of the highest level of public health and animal health protection as well as environmental protection. Otherwise, misuse (overuse of antibiotics) will lead to the spread of resistant pathogens, generally in animals, plants, and the environment and ultimately also in humans, representing one of the most serious global threats to public health.

The new legal regulation in the European Union does not limit the treatment of sick animals by administering them antibiotics, but its *ratio legis* excludes prophylactic treatment of the entire herd, with only a few diagnosed sick animals in the group. In other words, the EU legislator prohibits the massive and preventive use of antibiotics, e.g., in animal feed, water, etc. only for economic purposes. It should also be remembered that only effective supervision and control activities as well as monitoring studies of individual public administration bodies in all EU Member States will allow for a reliable assessment of the legitimacy and correctness of the use of antibiotics, and thus guarantee the protection of consumers against the consequences of their improper use.

The observation of the practice proves that in addition to the appropriate legal tools and organizational solutions, including the development of the principles of rational and safe use of antibiotics, the appropriate staffing of government and local government administration bodies to perform the above-mentioned duties is of key importance. Otherwise, staff shortages, insufficient level of financing, and high workloads will make the above-mentioned tasks ineffective or perhaps more accurately simulated, due to staff fluctuations.

## 6. Conclusions

The greatest discovery of the 20th century was the control of many diseases, both animal and human with antibiotics. Unfortunately, the overuse of antibiotics has resulted in the appearance of drug-resistant pathogenic bacteria in veterinary practice and medicine. The main goal of large-scale livestock breeders is to intensify production and reduce mortality in the herd. An easy way to do this is to use antibiotics. Increased animal production keeps animals crowded, which facilitates the transmission of various diseases. As shown in the review, the use of probiotics on farms can naturally bring about a balance of gut microbes and reduce the growth of pathogens in broiler, pig, piglet, and cattle slaughter farms. Based on the review of the latest articles from 2021, an increase in interest in the effects caused by *B. subtilis* can be noticed (Table 1). The results indicate that it could be a substitute for antibiotics and an effective growth promoter in broiler breeding [55,56,57,58]. The use of probiotics is of increasing importance for the alleviation of weight and immunity deficiencies as well as for lowering the mortality ratio in broiler farms [117]. The discussed studies show the positive effect of LAB and its metabolites on the welfare of farm animals. The limitations of antibiotics in farms have a positive effect on the environment and living organisms, including humans. The application of the new regulations in the law, which will come into force in 2022, is to limit completely the use of antibiotics for disease prevention before they appear in the herd. This is the next step to reduce the occurrence of resistance effects among pathogenic bacteria.

## Figures and Tables

**Table 1:** Overview of the application of beneficial bacteria in poultry farming in an *in vivo* study.

Probiotic/Postbiotic/Synbiotic	Form/Way of Administration	Effect	References
*Lactiplantibacillus plantarum* LTC-113	vaccination	Protection against *Salmonella* Typhimurium; stabilizing intestinal epithelial barrier	[28]
*Lactobacillus johnsonii*	*per os* in feed	Reduction of *Salmonella sofia* and *Clostridium perfringens*	[29]
*Ligilactobacillus salivarius* 59 and *Enterococcus faecium* PXN33/probiotic	*per os* in feed	Decreased colonization by *Salmonella* Enteritidis S1400	[30]
*E. faecium*/probiotic	*per os* in feed	Increased egg weight, serum FSH * levels, decreased *Bacteroidetes*	[31]
*Bacillus subtilis* C-3102/probiotic	*per os* in feed	Reduction of *Salmonella eteric* serovar *enteritidis* LM-7	[34]
*Pediococcus acidilactici*	*per os* in feed	Reduction of *Salmonella enterica* serovar Gallinarum	[35]
*Lactobacillus acidophilus* NCFM, *Lactobacillus crispatus* JCM 5810, *Lactobacillus gallinarum* ATCC 33199, and *Lactobacillus helveticus* CNRZ32	*per os* in feed	Inhibition of the growth of *Campylobacter jejuni*	[38]
*B. subtilis* MORI 91, *Clostridium butyricum* M7 and *L. plantarum* K34/commercial probiotic mix	*per os* in feed	Lowered rate of *E. coli*; positive modulation of the intestinal microbiota	[41]
Nisin/postbiotic	*per os* in feed	Natural growth promotor; positive influence on the modulation of the intestinal microbiota; limitation of pathogens in the gut	[42,46]
Chinese medicinal plants and *B. subtilis, L. acidophilus* (probiotic)	*per os* in water	Inhibition of *E. coli*; reduction of mortality; improvement BWG*	[48]
*L. plantarum* ŁOCK 0860, *L. reuteri* ŁOCK 1092, *L. pentosus* ŁOCK 1094, *Saccharomyces cerevisiae* ŁOCK 0119, *L. rhamnosus* ŁOCK 1087, *L. paracasei* ŁOCK 1091 and 2% inulin (synbiotic)	*per os* in feed	Increase of *Bifidobacterium* sp. and *Lactobacillus* sp.; decrease in the level of *Clostridium* sp. and *E. coli*	[27]
*L. plantarum* CCTCC M2016259, *Paenibacillus polymyxa* CGMCC1.1711	*per os* in feed	The positive effect of intestinal health and intestinal microbiota, improved body weight and feed conversion in *C. perfringens*-infected; increased intestinal SCFAs * levels	[52,53]
*L. acidophilus*	*per os* in feed	Increased body weight; reduced mortality; improvement of the immune response in *E. coli* O157-challenged chickens	[54]
*B. subtilis*	*per os* in feed	Increased FCR*, villus height to crypt depth ratio, and number of *Blautia*, *Faecalibacterium*, *Flavonifractor*, *Hydrogenoanaerobacterium*, and *Romboutsia*; decreased *Odoribacter;* improvement intestinal microbial composition	[55]
*B. subtilis* PB6	*per os* in feed	Increased calcium and phosphorus in plasma; increased bone mass and meat quality; improvement production and welfare	[56]
*B. subtilis* QST713	*per os* in feed	Increased level of *Lactobacillus* spp.; decreased level of *E. coli* and *Enterococcus* spp.; elongated villi; fewer deep crypts	[57]
*B. subtilis* DSM 29784	*per os* in feed	Increased FCR*, numbers of goblet cells, and superoxide dismutase activities in the jejunal mucosa; elongated villi	[58]
		Improved health, weight, the tight junction complex in necrotic enteritis-challenged broilers; increased numbers of *Butyricicoccus* and *Faecalibacterium* in the intestine; raised expression of INF-γ and IL-12	[59]
*E. faecium* PNC01	*per os* in feed	Inhibition of the growth of *Salmonella typhimurium*; elongated villi; reduced the length of jejunum and ileum; increased number of *Firmicutes* and *Lactobacillus*; reduced the number of *Bacteroides*	[60]

* FSH—follicle-stimulating hormone; BWG—body weight gain; SCFA—short-chain fatty acids; FCR—feed conversion ratio.

**Table 2:** Overview of the application of beneficial bacteria in pig and piglet farming in an *in vivo* study.

Strains	Form/Way of Administration	Effect	References
Okara fermented soy milk with *L. delbrueckii* subsp. *delbrueckii* TUA4408L/probiotic	*per os* ^1^	Better meat quality and growth performance; increased level of lactobacilli and *Lactococcus*	[68]
*L. delebureckii* CCTCC M 207040/probiotic	*per os* in feed ^1^	Reduced crypt depth in the jejunum and ileum; increased gut integrity	[69]
*C. butyricum* ZJU-F1 and *B. licheniformis*/probiotic			[70]
*L. salivarius* MP100/probiotic	*per os* ^1,3^	Antagonistic activity against *C. perfringens* MP34, *E. faecalis* MP42, *S. aureus* MP83, *Streptococcus suis* MP205, *Trueperella pyogenes* MP214, *E. coli* MP73 (F4) and MP77 (F18), *S.* Typhimurium MP55, and *Klebsiella pneumoniae* MP87	[71]
*Lactobacillus gasseri* LA39 and *Limosilactobacillus frumenti*/probiotic	feces and saline solutions/*per os* ^2^	Prevent diarrhea after weaning	[72]
*L. salivarius* 144 and 160/probiotic	*per os* ^1^	Increased level of lactobacilli and reduction level of *Bacteroides* and *Fibrobacter* in the gastrointestinal tract; reduced diarrhea	[73]
*L. plantarum* SC01/probiotic	Microcapsulation/*per os* ^2^	Antagonistic activity against *E. coli*, *S. aureus*, *B. subtilis*, *Salmonella* sp., and *L. monocytogenes*	[74]
*L. plantarum* 22F and 25F, and *P. acidilactici* 72N/probiotic	Spray drying microencapsulation/*per os* ^2^	Improvement of intestinal integrity, elongation of intestinal villi in the jejunum, the appearance of microorganisms positively influencing the intestinal microbiome, and improved growth of individuals in the rearing cycle	[75]
*L. johnsonii* XS4/probiotic	Freeze-dried/*per os* ^3^	Increased number of weaned piglets increased litter weight	[76]
*Limosilactobacillus reuteri* P7, *Lactobacillus amylovorus* P8, and *L. johnsonii* P15/probiotic	*per os* ^3^	Positive effect on the reproductive performance of sows and the growth of weaned piglets and reduced the occurrence of diarrhea	[77]
*E. faecium* DSM 7134	*per os* ^3^	Reduced level of *E. coli* in feces after weaning the piglets	[78]
*L. delbrueckii* subsp. *bulgaricus*, *L. rhamnosus*, *L. acidophilus*, *L. plantarum*, *Streptococcus salivarius* subsp. *thermophilus*, *Bifidobacterium bifidum*, *E. faecium*, *Candida pintolopesii*, and *Aspergillus oryzae*/probiotic	*per os* ^1,3^	Increased concentration of acetic, propionic, and butyric acids in the feces	[79]
*L. plantarum* CAM6/probiotic	*per os* ^3^	Improved nutritional value of milk	[80]
*B. subtilis* CW4 and *E. faecium* CWEF/probiotic	*per os* ^1,3^	Improvement in the quality of sows’ milk; better immunity at sows	[84]
*L. reuteri* 1/probiotic	freeze-dried/*per os* ^2^	Increased carcass yield; improved meat quality and flavor	[88]
*B. subtilis* WB800	*per os* ^1^	Enhanced respiratory immunity	[89]

The experiment was carried out on 1—piglets, 2—pigs, 3—sows.

**Table 3:** Overview of the application of potential probiotic bacteria in bovine farming in an *in vitro* study.

Probiotic/Postbiotic	Form/Way of Administration	Effect	References
Nisin derivatives/postbiotic	Solution	Antibacterial activity against *S. aureus*	[101]
Bovicin HC5/postbiotic	Solution	Bactericidal effect against *L. monocytogenes*, *Salmonella* Typhimurium, and some species of *Clostridium* and *Bacillus*	[103,104]
*L. lactis* LL11 and SL153/postbiotic	Supernatant	Bactericidal effect against the most common occurring mastitis pathogen	[106]
Bacteriocin from *L. lactis* CJNU 3001/postbiotic	Bacteriophage	Inhibition of the growth of *S. aureus* KCTC 3881	[108]
*Lactobacillus gasseri*, *Limosilactobacillus reuteri*, and *Ligilactobacillus salivarius*/postbiotic	Supernatant	Bactericidal activity against *Escherichia coli* O157:H7, *Mycobacterium avium* ssp. *paratuberculosis*, and *Salmonella* species	[112]
*L. gasseri* LA806	Live and heat-inactived	Inhibition of the growth of *S. aureus;* barrier and immunomodulatory	[113]

## Data Availability

Not applicable.
